# The relationship between obstructive sleep apnea and visual hallucinations in PD patients: a polysomnography study

**DOI:** 10.3389/fneur.2023.1275660

**Published:** 2024-01-11

**Authors:** Jun Zhu, Yang Zhao, Yinyin Jiang, Yang Pan, Xu Jiang, Yaxi Wang, Dongfong Li, Li Zhang

**Affiliations:** Department of Geriatric Neurology, Nanjing Brain Hospital Affiliated to Nanjing Medical University, Nanjing, Jiangsu, China

**Keywords:** Parkinson's disease, visual hallucinations, cognition, sleep, obstructive sleep apnea, apnea-hypopnea index

## Abstract

**Purpose:**

Parkinson's disease (PD) patients frequently experience visual hallucinations (VHs) and obstructive sleep apnea (OSA). The aim of this study was to describe the prevalence and clinical correlates of VHs and OSA in the Chinese population with PD.

**Materials and methods:**

A sample of 489 PD patients was recruited for the present study. Patients were categorized as having formed VHs (FVHs) or minor VHs (MVHs) or as non-hallucinators (NVHs) according to the Unified Parkinson's Disease Rating Scale (UPDRS) and an initial questionnaire. Polysomnography (PSG) was used for objective assessment of sleep.

**Results:**

VHs were observed in 143 (29.2%) patients. Among them, 75 of the hallucinators experienced MVHs, and 68 experienced FVHs. The disease duration, UPDRS Part III score, Hoehn and Yahr (H–Y) stage, Pittsburgh Sleep Quality Index (PSQI) score and rapid eye movement (REM) sleep behavior disorder (RBD) score of hallucinators were significantly greater than those of non-hallucinators (*P* < 0.05). We also observed OSA in 38.7, 54.7, and 63.3% of the NVH, MVH, and FVH groups, respectively. PSG showed that the VH groups had a lower total sleep time, lower sleep efficiency, higher arousal index, lower sleep latency, lower N1%, higher apnea-hypopnea index (AHI), higher average duration of apnea, higher respiratory-related arousal (RRA), and lower values of the lowest O_2_ and mean O_2_. The forward binary logistic regression model showed that AHI, N1%, RRA and lowest O_2_ were independently associated with VHs in PD patients.

**Conclusions:**

Our results confirm the high prevalence of VHs and OSA as well as their relationship in patients with PD.

## Introduction

Parkinson's disease (PD) is the second most prevalent neurodegenerative disorder globally, and it affects ~10 million individuals (1). This disease is a progressive brain disorder that affects elderly individuals and is associated with multiple factors, including mitochondrial dysfunction that leads to dopaminergic system degeneration (2). Although PD is a movement disorder, it is accompanied by a wide range of non-motor symptoms, such as neuropsychiatric manifestations, an abnormal sense of smell, urinary symptoms, sleep disorders, autonomic dysfunction, and sensory disorders (3).

The most prevalent neuropsychiatric symptom of PD is visual hallucinations (VHs) (4). Our previous study including 371 patients with primary PD found that 19.4% of patients had VHs (5). Types of VHs include formed visual hallucinations (FVHs) and minor visual hallucinations (MVHs). FVHs often appear in dim light, especially when an individual is falling asleep, and mainly consist of people and animals, which often appear suddenly in colorful and blurry forms. MVHs consist of presence hallucinations, passage hallucinations, and visual illusions (6). Numerous studies have indicated that VHs are linked with cognitive decline and elevated mortality in PD patients (7).

There is a growing interest in the impact of sleep and sleep disturbances on the brain, particularly in connection with aging and PD. Due to mouth breathing, pharyngeal collapse, motor coordination impairment, and autonomic dysfunction, PD patients have a high prevalence of obstructive sleep apnea (OSA) (8). OSA is characterized by recurrent hypopnea or apnea during sleep due to airway obstruction, accompanied by headache, a dry mouth in the morning, lack of concentration, excessive daytime sleepiness and various neurocognitive, and psychological manifestations (9).

However, the association between VHs and OSA in PD patients is unclear. The objective of this study was to examine the relationship between VHs and OSA through a retrospective cohort study conducted in the Chinese PD population.

## Materials and methods

### Population

**Figure 1:**
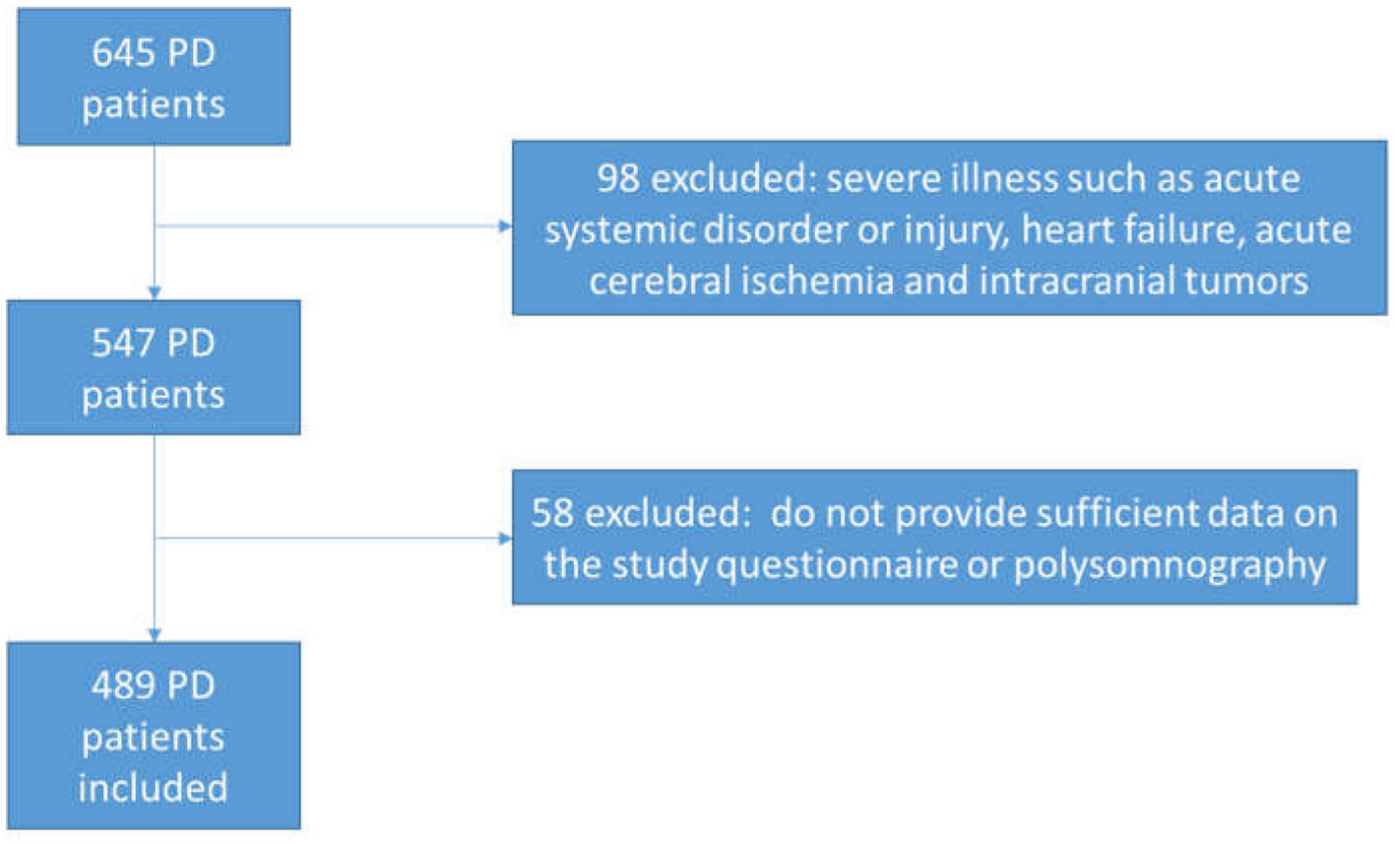
Flowchart for PD patient selection.

Between January 2021 and July 2023, a total of 489 consecutive PD patients were recruited from the Department of Geriatrics, Nanjing Brain Hospital. All patients with PD met the criteria set by the United Kingdom Parkinson's Disease Society Brain Bank (10) (Figure 1). The exclusion criteria were as follows: (1) diagnoses of progressive supranuclear palsy, multiple system atrophy, Lewy body dementia, corticobasal degeneration, or other forms of atypical parkinsonism; (2) severe illness, such as acute systemic disorder or injury, heart failure, acute cerebral ischemia or intracranial tumors; or (3) missing questionnaire or polysomnography (PSG) data.

### Procedures

To obtain the demographic and clinical characteristics of each patient, including age, sex, age of disease onset, disease duration, education level, body mass index (BMI) and use of antiparkinsonian drugs, a structured questionnaire was utilized. Levodopa equivalent doses (LEDs) were calculated based on previously published data (11).

Clinical assessments of the PD patients, including their scores on the Unified Parkinson's Disease Rating Scale (UPDRS) (12) and their Hoehn and Yahr (H-Y) stage, were used to evaluate PD motor symptoms and disease severity. The Montreal Cognitive Assessment (MoCA) was utilized to evaluate cognitive function (13). The Parkinson's Disease Sleep Scale (PDSS) and Pittsburgh Sleep Quality Index (PSQI) were used to evaluate the overall sleep quality of patients (14, 15). The STOP-Bang scale and Epworth Sleepiness Scale (ESS) were used to assess the risk of apnea and daytime drowsiness of patients, respectively (16, 17). The Rapid Eye Movement Sleep Behavior Disorder Screening Questionnaire (RBD-SQ) was used to assess rapid eye movement (REM) sleep behavior disorder (RBD) in PD patients (18). The Hamilton Depression Rating Scale (HAMD), which includes 24 items, and the Hamilton Anxiety Rating Scale (HAMA) were utilized to quantify the severity of depression and anxiety, respectively (19, 20). We used the UPDRS Part 1, Question 2 to conduct preliminary screening for hallucinatory symptoms. This item was scored on the following scale: 0 = absence of VHs, 1 = illusions or non-formed hallucinations (e.g., MVHs), 2 = FVHs without loss of insight, 3 = FVHs with loss of insight, and 4 = delusions or paranoia. We also created a questionnaire that contained 11 items covering the nature and properties of the patients' VHs (5).

### Polysomnography

The full-night, attended, laboratory-based video PSG data were recorded on a Compumedics Grael-HD 64 (Compumedics Grael-HD, Australia) polygraph. The PSG data were interpreted according to the American Academy of Sleep Medicine (AASM) Sleep Scoring Manual (21). The signal sampling frequency was set to 256 Hz, and the notch frequency was 60 Hz. The bandpass filter was set to 3–30 Hz. The low-pass frequency of the EMG channel was set to 10 Hz. Electrodes were placed according to the international 10–20 system, and data were recorded from a total of 19 channels: 6 EEG channels (F3-M2, F4-M1, C3-M2, C4-M1, O1-M2, O2-M1), 2 electrooculography (EOG) channels, 1 chin electromyography (EMG) channel, 1 snoring channel, 1 oxygen flow channel, 1 nasal thermosensitive channel, 1 chest respiratory movement channel, 1 abdominal respiratory movement channel, 1 oxygen saturation channel, 1 postural channel, and 2 lower limb EMG channels. PSG data were recorded between 23:00 and 07:00. Sleep efficiency (SE), sleep latency (SL), non-REM (NREM) sleep stage 1 (N1) time (min), NREM sleep stage 3 (N3) time (min), total sleep time (TST; min), the periodic limb movement index (PLMI), the apnea-hypopnea index (AHI), mean O_2_, lowest O_2_, the average duration of apnea, and respiratory-related arousal (RRA) were all recorded. The density of REMs was determined by counting the number of eye movements per minute during REM sleep over the entire night (22). OSA was defined as upper airway collapse resulting in a decrease (hypopnea) or loss (apnea) of airflow for at least 10 s. The severity of OSA was defined using the AHI proposed by the AASM. The severity levels were categorized as follows: mild (AHI = 5–15), moderate (AHI = 15–30), and severe (AHI >30).

### Statistical analysis

All continuous data are presented as the mean ± standard deviation, and categorical variables are represented as percentages. To compare continuous data, Student's *t*-test was utilized, while chi-square tests were used to analyze associations between categorical variables. Differences in continuous variables were evaluated by conducting an analysis of variance (ANOVA) with a *post-hoc* analysis employing Punnett's T3 and Scheffe's tests. To explore the potential clinical factors associated with VHs, a forward binary logistic regression model using VHs as the dependent variable and disease characteristics identified as significant in the univariate analysis as independent covariables was employed for a multivariate analysis. All analyses were conducted using SPSS 19.0 (SPSS Inc., Chicago, IL).

## Results

### Sociodemographic and clinical characteristics of patients with and without hallucinations

Table 1 presents the demographic characteristics of the patients with and without hallucinations. VHs were observed in 143 patients (29.2%). Regarding the typical VHs, 68 (13.9%) patients described their VHs as a complex visual image (i.e., FVHs) and 75 (15.3%) patients described their VHs as MVHs. No differences were found between patients with and without hallucinations in terms of age, sex, BMI, age of disease onset, or education level. Patients with FVHs had a longer disease duration, more severe motor symptoms, a higher H-Y stage, and a higher LED than patients with MVHs or those without hallucinations (all *P* < 0.05). We divided PD patients into three groups based on their motor symptoms, namely, the tremor dominant group, akinetic rigid dominant group, and mixed motor group, accounting for 52, 26, and 22% of patients, respectively. We compared VHs among the three groups. We found that the akinetic rigid dominant group and mixed motor group had more frequent and severe hallucinatory symptoms.

**Table 1:** Demographic characteristics of enrolled patients with Parkinson's disease according to the presence of VHs.

	**PD-NVHs**	**PD-MVHs**	**PD-FVHs**	***t*/χ^2^**	***P***
	***N*** = **346 (70.8%)**	***N*** = **75 (15.3%)**	***N*** = **68 (13.9%)**		
Age, y, mean ± SD	63.49 ± 7.16	64.41 ± 10.35	65.37 ± 8.12	1.357	0.260
Sex (male, %)	190/156 (54.9%)	45/30 (60.0%)	36/32 (52.9%)	0.097	0.755
BMI, kg/m^2^	23.74 ± 3.35	23.80 ± 3.38	24.91 ± 4.34	0.692	0.559
Duration, y	5.91 ± 3.99	6.19 ± 4.47^ab, bc^	7.19 ± 4.21^ac, bc^	2.987	**0.022** ^*^
Age of disease onset, y	58.47 ± 10.34	59.08 ± 10.08	60.21 ± 7.08	0.087	0.796
Education, y	9.36 ± 5.23	10.03 ± 5.98	8.03 ± 5.35	0.543	0.656
UPDRS-III score	20.06 ± 11.33	24.3 ± 8.99^ab, bc^	27.06 ± 11.33^ac, bc^	3.978	**0.013** ^*^
H-Y stage	1.82 ± 0.91	2.31 ± 0.72^ab^	2.55 ± 0.54^ac^	1.565	**0.043** ^*^
LED (mg/d)	299.42 ± 224.43	320.60 ± 160.68^ab^	328.60 ± 140.73^ac^	1.674	0.039^*^
PD phenotype				15.14	**0.004** ^*^
Tremor dominant	197 (56.9%)	32 (42.7%)	25 (36.8%)		
Akinetic rigid dominant	75 (21.7%)	25 (33.3%)	27 (39.7%)		
Mixed motor subtype	74 (21.4%)	18 (24.0%)	16 (23.5%)		

PD, Parkinson's disease; NVHs, non-visual hallucinations; MVHs, minor visual hallucinations; FVHs, formed visual hallucinations; BMI, body mass index; LED, levodopa equivalent doses.

^*^Significant difference.

^ab^Significant difference between the NVH and MVH groups.

^bc^Significant difference between the MVH and FVH groups.

^ac^Significant difference between the NVH and FVH groups.

Bold values indicate the significant difference.

### Comparison of non-motor symptoms between individuals with and without hallucinations

Non-motor symptoms (NMSs) were also compared between the patients with and without hallucinations. The differences in NMSs are shown in Table 2. Patients with FVHs had significantly higher scores on the STOP-Bang scale, ESS, PSQI, and RBD-SQ (all *P* < 0.01), indicating that sleep disorders were more common in the patients with hallucinations. The patients with hallucinations had worse scores on the MoCA overall and in the domains of visuospatial/executive function, abstraction and orientation than those of patients with MVHs or patients without hallucinations (all *P* < 0.05). There were no differences in HAMA or HAMD scores among the three groups.

**Table 2:** NMSs of enrolled patients with Parkinson's disease according to the presence of VHs.

	**PD-NVHs**	**PD-MVHs**	**PD-FVHs**	***t***	***P***
	***N*** = **346 (70.8%)**	***N*** = **75 (15.3%)**	***N*** = **68 (13.9%)**		
STOP-Bang scale score	2.06 ± 0.35	3.31 ± 0.16^ab, bc^	4.24 ± 0.32^ac, bc^	5.987	**<0.001** ^*^
ESS score	5.21 ± 0.31	6.31 ± 0.24^ab^	6.56 ± 0.19^ac^	4.768	**<0.001** ^*^
MoCA score	26.65 ± 3.56	21.15 ± 2.95^ab^	19.14 ± 1.95^ac^	2.787	**<0.001** ^*^
Visuospatial/executive	3.58 ± 1.34	2.25 ± 1.60	2.46 ± 1.60	3.143	**<0.001** ^*^
Naming	2.79 ± 0.52	2.35 ± 0.62	2.06 ± 0.35	2.087	0.453
Attention	5.56 ± 0.70	5.56 ± 0.70	2.51 ± 0.74	1.892	0.345
Language	2.61 ± 0.64	2.61 ± 0.64	1.93 ± 0.48	2.278	0.124
Abstraction	1.64 ± 0.59	1.21 ± 0.59^ab^	1.19 ± 0.64^ac^	2.154	**0.002** ^*^
Delayed memory	3.29 ± 1.42	2.38 ± 1.44	2.29 ± 1.44	1.986	0.453
Orientation	5.95 ± 0.27	4.87 ± 0.38^ab^	3.95 ± 0.27^ac^	2.034	**<0.001** ^*^
PDSS score	120.23 ± 21.39	104.23 ± 19.79^ab, bc^	96.13 ± 14.23^ac, bc^	7.233	**<0.001** ^*^
PSQI score	8.87 ± 3.12	12.31 ± 5.43^ab, bc^	15.84 ± 3.23^ab, bc^	6.134	**<0.001** ^*^
HAMA score	4.61 ± 9.29	4.57 ± 2.13	7.43 ± 4.27	0.533	0.234
HAMD score	6.43 ± 6.37	6.48 ± 4.25	8.43 ± 4.36	0.787	0.543
RBD-SQ score	3.13 ± 1.37	5.21 ± 2.21^ab^	6.03 ± 1.22^ac^	**1.987**	**0.021** ^*^

PD, Parkinson's disease; NVHs, nonvisual hallucinations; MVHs, minor visual hallucinations; FVHs, formed visual hallucinations; MoCA, Montreal Cognitive Assessment; PDSS, PD Sleep Scale; PSQI, Pittsburgh Sleep Quality Index; HAMA, Hamilton Anxiety Rating Scale; HAMD, Hamilton Depression Rating Scale; RBD-SQ, RBD Screening Questionnaire.

The P-value was adjusted for disease duration, the UPDRS Part III score, and the H-Y stage.

^*^Significant difference.

^ab^Significant difference between the NVH and MVH groups.

^bc^Significant difference between the MVH and FVH groups.

^ac^Significant difference between the NVH and FVH groups.

Bold values indicate the significant difference.

### Comparison of sleep parameters between patients with and without hallucinations

Between-group differences in parameters are shown in Table 3. Individuals with hallucinations also had a lower TST, a lower SE, a higher arousal index, a longer SL, a higher percentage of time in N1 out of TST (N1%), a lower percentage of time in N3 (N3%), a higher risk of RBD and a higher REM density. This means that patients without hallucinations had deeper and more continuous sleep. The REM sleep latency, percentage of time in N2 (N2%), and REM sleep percentage did not show any statistically significant differences among the three groups.

**Table 3:** Sleep parameters in PD patients with and without VHs.

	**PD-NVHs**	**PD-MVHs**	**PD-FVHs**	***t*/*x***	***P***
	***N*** = **346 (70.8%)**	***N*** = **75 (15.3%)**	***N*** = **68 (13.9%)**		
Total sleep time (min)	358.87 ± 87.12	324.97 ± 98.01^ab, bc^	299.97 ± 98.01^ac, bc^	4.516	**0.012** ^*^
Sleep efficiency (%)	80.57 ± 16.23	73.75 ± 14.72 ^ab, bc^	68.75 ± 12.45^ac, bc^	9.374	**<0.001** ^*^
Arousal index	10.30 ± 11.01	20.81 ± 10.14^ab, bc^	25.81 ± 10.14^ab, bc^	5.863	**0.007** ^*^
Sleep latency (min)	42.19 ± 18.17	56.19 ± 23.17^ab, bc^	75.31 ± 17.83^ab, bc^	8.987	**<0.001** ^*^
REM sleep latency (min)	111.53 ± 39.48	109.21 ± 37.14	112.51 ± 37.14	0.697	0.550
N1%	9.11 ± 7.48	14.36 ± 5.39^ab, bc^	21.36 ± 5.39^ac, bc^	10.134	**<0.001** ^*^
N2%	48.34 ± 15.46	53.48 ± 12.41	51.38 ± 16.33	0.567	0.734
REM sleep%	16.95 ± 10.26	15.02 ± 12.48	16.23 ± 12.89	0.378	0.302
N3%	18.02 ± 6.21	14.83 ± 6.81^ab, bc^	8.83 ± 6.73^ab, bc^	6.159	**0.004** ^*^
REM density	8.57 ± 4.16	12.41 ± 3.56^ab^	13.23 ± 4.85^ac^	4.765	**0.018** ^*^
RBD (%)	128 (36.9%)	42 (56.0%)^ab^	39 (57.3%)^ac^	4.892	**0.013** ^*^
PLMI	9.87 ± 4.31	12.82 ± 3.37^ab^	13.82 ± 2.35^ac^	3.211	**0.023** ^*^

PD, Parkinson's disease; NVHs, non-visual hallucinations; MVHs, minor visual hallucinations; FVHs, formed visual hallucinations; N1%, NREM sleep stage 1, % of total sleep time; RBD, RBD Screening Questionnaire; PLMI, periodic limb movements index.

The P-value was adjusted for disease duration, the UPDRS Part III score, and the H-Y stage.

^*^Significant difference.

^ab^Significant difference between the NVH and MVH groups.

^bc^Significant difference between the MVH and FVH groups.

^ac^Significant difference between the NVH and FVH groups.

Bold values indicate the significant difference.

### Comparison of OSA parameters between patients with and without hallucinations

The prevalence rates of OSA (AHI > 5) in patients with FVHs, MVHs, and without hallucinations were 63.3, 54.7, and 38.7%, respectively (Table 4). A higher proportion of patients with FVHs had moderate to severe OSA (AHI > 15). These patients also experienced more severe hypoxia, a longer average duration of apnea and a higher RRA (all *P* < 0.05).

**Table 4:** OSA sleep parameters in PD patients with and without VHs.

	**PD-NVHs**	**PD-MVHs**	**PD-FVHs**	***t*/χ^2^**	***P***
	***N*** = **346 (70.8%)**	***N*** = **75 (15.3%)**	***N*** = **68 (13.9%)**		
Apnea-hypopnea index	5.70 ± 2.66	9.11 ± 3.62	12.21 ± 4.59	12.516	**<0.001** ^*^
<5	212 (61.3%)	34 (45.3%)	25 (36.7%)	9.876	**<0.001** ^*^
5–15	58 (16.7%)	11 (14.6%)	8 (11.7%)		
15–30	52 (15.2%)	19 (25.3%)	22 (32.5%)		
>30	24 (6.9%)	11 (14.6)	13 (19.1%)		
Mean O_2_ (%)	95.57 ± 16.23	91.75 ± 14.72^ab, bc^	87.75 ± 12.45^ac, bc^	4.374	**<0.001** ^*^
Lowest O_2_ (%)	83.30 ± 11.01	72.81 ± 10.14^ab, bc^	65.81 ± 10.14^ac, bc^	8.863	**<0.001** ^*^
Average duration of apnea(s)	21.53 ± 39.48	36.51 ± 37.14^ab, bc^	43.51 ± 37.14^ac, bc^	7.697	**<0.001** ^*^
Respiratory related arousal	9.11 ± 7.48	12.30 ± 5.49^ab, bc^	20.29 ± 5.37^ac, bc^	10.134	**0.023** ^*^

PD, Parkinson's disease; NVHs, non-visual hallucinations; MVHs, minor visual hallucinations; FVHs, formed visual hallucinations.

The P-value was adjusted for disease duration, the UPDRS Part III score, and the H-Y stage.

^*^Significant difference.

^ab^Significant difference between the NVH and MVH groups.

^bc^Significant difference between the MVH and FVH groups.

^ac^Significant difference between the NVH and FVH groups.

Bold values indicate the significant difference.

### Factors independently correlated with visual hallucinations

The results of the multivariate logistic regression analysis are summarized in Table 5. VHs were independently associated with a higher AHI [odds ratio (OR), 9.382; 95% confidence interval (CI), 4.534–70.796], more severe RRA [OR, 4.382; 95% CI, 2.234–41.420], lower lowest O_2_ [OR, −2.186; 95% CI, 1.067–4.154], and higher N1% [OR, 2.223; 95% CI, 1.108–5.638].

**Table 5:** Clinical factors correlated with hallucinations in PD patients.

	***OR***	**95% CI**	***P* ^a^**
AHI	9.382	2.534–160.796	**<0.001** ^*^
Respiratory related arousal	4.382	4.534–80.796	**<0.001** ^*^
Lowest O_2_ (%)	−2.186	0.067–0.034	**0.007** ^*^
N1%	2.223	0.108–0.638	**0.013** ^*^

AHI, apnea-hypopnea index; OR, odds ratio; CI, confidence interval.

^a^The P-value is calculated from a forward binary logistic regression analysis.

^*^Significant difference.

Bold values indicate the significant difference.

## Discussion

VHs are the most common neuropsychiatric symptoms in patients with PD. The mechanism behind their formation is complex, and their clinical manifestations are diverse, making it easy for VHs to be overlooked. A previous study showed that between 8 and 40% of patients with PD had VHs (23). In our study, 13.9% of the FVHs reported were complex visual images that typically moved and were of normal size, lasting for seconds to minutes. On the other hand, our findings also indicated that minor or simple hallucinations (e.g., MVHs), which affect 15.9% of all PD patients, are generally less likely to be apparent to informants and hence to be underestimated by them. These minor phenomena typically lasted for a brief period and occurred more than once a week, while insight was completely preserved. We further discovered that patients with FVHs had a longer duration of illness and more severe motor symptoms than those with MVHs and NVHs. The pathophysiology of VHs is a complex matter. It has been suggested that injuries to the dopaminergic, serotonergic, and cholinergic systems in various brain areas may play a role in causing hallucinations (24). As the disease progresses, the loss of these neurotransmitters may worsen hallucinatory symptoms. Nonetheless, we discovered that the severity of PD was not significantly independently associated with VHs, indicating that physicians ought not to rely solely on conventional measures of disease severity to identify patients at risk of developing VHs.

There is still a debate as to whether anti**-**PD medications contribute to the development of VHs. In our study, we found that patients with VHs had higher LEDs than those without hallucinations. Benzhexol, amantadine, levodopa, catechol-O-methyltransferase inhibitors, and MAO-B inhibitors contributed to VHs. However, anticholinergic agents may cause hallucinations and are not included in LED calculations. Due to the potential for cognitive and urinary side effects as well as other side effects, Parkinson's disease patients seldom use anticholinergic drugs, which is why these drugs were not included in this study. In the future, we will conduct a more detailed calculation of the proportion and dosage of each type of anti-Parkinson's medication used by individuals with and without VHs.

Our study is the first to compare the NMSs among three groups of PD patients (with FVHs, with MVHs and without VHs). The relationship between depression/anxiety symptoms and VHs is still unknown. Fénelon et al. reported a higher prevalence of depression in patients with VHs (25), and Mack et al. (26) found that patients with VHs had severe depressive symptoms. However, some longitudinal studies that focused on VHs did not report a clear relationship with depression, anxiety, or apathy in PD patients (27, 28). We conducted an evaluation using the HAMA and HAMD and found no statistically significant differences in scores on these scales between patients with and without VHs. In the univariate analysis, we found that the PD patients with VHs showed more severe cognitive impairment, especially in visuospatial/executive function, abstraction, and orientation, than the PD patients without VHs, especially those in the FVH group, consistent with Tom's study showing that VHs are probably the major independent predictor of cognitive deterioration (29). However, neither emerged as an independent factor predictive of VHs in our study. The association between cognitive impairment and VHs could be due to a parallel pathophysiological process, and these symptoms may represent a common endpoint of neurodegeneration. It is unclear how the pathologic process is related to independent cognitive changes and VHs.

The relationship between VHs and RBD is still unknown. In this study, we used PSG data to diagnose RBD to investigate this relationship. The prevalence rates of RBD in the group without VHs (the NVH group), MVH group, and FVH group were 36.9, 56.0, and 57.3%, respectively. The prevalence of RBD in patients with VHs was significantly higher than that in the NVH group, but there was no difference between the MVH and FVH groups. An 8-year longitudinal study found that RBD was correlated with the occurrence of hallucinations, regardless of disease duration, motor stage, age or sex (30). An association between VHs and RBD was also demonstrated in a study by Gjerstad et al. (31). Cholinergic dysfunction could be the common pathway for both RBD and VHs (32). We also found that REM density was higher in VH patients, which means that patients with VHs have more active eye movements during REM sleep. The mechanism underlying this difference is not yet clear, but an increased density of REM sleep may lead to greater activation of the motor cortex.

We further examined the correlation between sleep habits and VHs in patients with PD. Our data showed that patients with VHs had worse sleep disruption or poorer sleep quality as assessed by the PDSS, PSQI, and ESS. Consistent with Barnes's study (33), PD patients with hallucinations reported more sleep disturbances and unexpected daytime sleepiness than those without hallucinations. However, this difference was not previously confirmed by PSG data. Our investigation utilizing PSG demonstrated that patients without VHs had a considerably longer TST than those with VHs. Patients with VHs exhibited lower SE, a higher arousal index, more N1 sleep, less N3 sleep, and more periodic limb movements. We also confirmed an independent association between VHs and N1%. This implies that patients experiencing hallucinations also exhibited poor overall sleep quality, sleep continuity, and depth of sleep. Prolonged SL and greater sleep-arousal changes have been associated with a prolonged state of drowsiness, thereby increasing the likelihood of experiencing VHs (34). A deeper understanding of abnormal sleep patterns and VHs could be obtained through a longitudinal investigation of whether sleep disturbances precede VHs or whether VHs cause sleep disturbances.

OSA is a prevalent disorder that is characterized by recurrent upper airway obstruction during sleep, which results in intermittent hypoxemia and sleep fragmentation. It is estimated that 20–60% of PD patients have concomitant OSA (35). OSA patients with PD exhibit a lower desaturation index, lower mean and nadir oxygen saturation values, and longer time with an oxygen saturation below 90% throughout the night than OSA patients without PD (36, 37). Previous studies have shown an association between vascular comorbidities and OSA as well as the severity of motor and cognitive symptoms in PD (38). However, there is a lack of studies assessing the relationship between OSA and VHs. Approximately 44.6% of our PD patients had OSA (AHI>5], consistent with a previous study. We also found that the prevalence rates of OSA were 38.7, 54.7, and 63.3% in PD patients in the NVH, MVH, and FVH groups, respectively. As hallucinatory symptoms worsen, the accompanying OSA also became more severe. Patients with PD frequently exhibit sleep disorders, including drowsiness, insomnia, and RBD. These sleep problems, such as daytime drowsiness, nighttime snoring, and frequent apnea, are similar to the symptoms of OSA. OSA has the potential to worsen motor and cognitive impairments in PD patients by causing hypoxemia and reducing sleep quality, ultimately impacting motor control and neural conduction. Furthermore, the prevalence of OSA among PD patients is high, and it may be associated with muscle tone disorders, laryngeal muscle dysfunction, and abnormalities in the structure of the upper respiratory tract. Additional research is needed to determine the precise correlation between OSA and PD as well as the influence of OSA on PD symptoms.

To our knowledge, this is the first study to use PSG and to explore the association between OSA and VHs in patients with PD. We not only focused on the prevalence of OSA in each group but also explored parameters related to OSA, such as the mean O_2_, lowest O_2_, average duration of apnea, and RRA. We found that the AHI, RRA, and lowest O_2_ were independent risk factors for VHs. Patients with OSA frequently experience cognitive and emotional issues, such as reduced concentration, memory impairment, and emotional instability. These symptoms could be associated with hypoxia in the brain during sleep and its impact on neuronal activity. By utilizing fMRI, it would be possible to observe abnormal brain activity patterns during sleep and identify specific regions associated with cognitive and behavioral issues in OSA patients. For example, OSA patients exhibited a decrease in metabolic rates in the frontal, temporal, and parietal lobes, which are associated with cognitive control, memory, and emotion regulation (39). Furthermore, individuals with OSA may exhibit brain activity associated with abnormal sleep patterns, which may include reduced slow wave sleep and increased REM sleep. These changes in sleep structure may further impact brain function and cognitive performance, potentially resulting in VHs. The intermittent hypoxia and frequent arousal that occur with OSA may also play a significant role in the pathophysiology of psychosis, particularly the development of VHs. PSG or respiratory monitoring should be a routine method for monitoring PD patients with VHs.

This study also has some limitations. First, this study was a retrospective study and lacked follow-up data. We are currently gathering follow-up data for subsequent statistical analysis. Second, this study does not provide a comparison of the occurrence and severity of VHs before and after OSA treatment. In our subsequent studies, we will evaluate the presence of VHs before and after continuous positive airway pressure (CPAP) treatment.

## Conclusion

In conclusion, we found that the prevalence of VHs among PD patients was 29.2%. We further discovered that PD patients with FVHs experienced longer illness durations and severe motor symptoms. PD patients with VHs also exhibited NMSs such as cognitive dysfunction and RBD, and this group had a higher percentage of patients with sleep disorders, a lower TST, a lower SE and a longer SL. Our results confirmed that 44.6% of PD patients had OSA. We also demonstrated that the AHI, N1%, lowest O_2_, and RRA were significant predictors of VHs. Therefore, clinicians should be aware of the importance of screening for and managing OSA in PD patients, and future studies should concentrate on exploring the pathophysiology of these symptoms.

## Data availability statement

The raw data supporting the conclusions of this article will be made available by the authors, without undue reservation.

## Ethics statement

The studies involving humans were approved by Ethics Committee of Brain Hospital Affiliated to Nanjing Medical University (ethics approval number: 2021-KY007-01). The studies were conducted in accordance with the local legislation and institutional requirements. The participants provided their written informed consent to participate in this study.

## Author contributions

JZ: Investigation, Methodology, Writing—original draft. YZ: Data curation, Writing—review & editing. YJ: Data curation, Writing—review & editing, Validation. YP: Writing—review & editing. XJ: Writing—review & editing. YW: Writing—review & editing, Conceptualization, Data curation, Investigation. DL: Writing—review & editing. LZ: Funding acquisition, Project administration, Writing—review & editing.
